# Editorial for the Special Issue on Advanced Abrasive Processing Technology and Applications

**DOI:** 10.3390/ma19030599

**Published:** 2026-02-04

**Authors:** Yufei Gao

**Affiliations:** 1 Key Laboratory of High Efficiency and Clean Mechanical Manufacture of Ministry of Education, School of Mechanical Engineering, Shandong University, Jinan 250061, China; yfgao@sdu.edu.cn; 2 State Key Laboratory of Advanced Equipment and Technology for Metal Forming, Shandong University, Jinan 250061, China

## 1. Introduction for Special Issue of Advanced Abrasive Processing Technology and Applications

Materials such as semiconductor materials [1], engineering ceramics [2], optical glasses [3], laser crystals [4], superalloys [5], titanium alloys [6], and composite materials [7,8] have been widely applied in semiconductor chips, photovoltaic solar cells, advanced optics, and the aerospace, automotive, and communication sectors due to their superior mechanical properties and functional stability. Advanced abrasive processing technology has become an indispensable method and a critical technical pillar for the high-efficiency, high-quality, and high-precision manufacturing of critical components made from these materials. Moreover, the requirements for abrasive processing techniques are becoming increasingly stringent. For instance, driven by the demand for “cost reduction and efficiency enhancement,” photovoltaic silicon substrates are evolving toward thinner thicknesses and larger dimensions [9], which imposes higher requirements on the yield of the diamond wire sawing process [10]. Similarly, in the semiconductor industry, the thickness and uniformity of diamond wire-sawn wafers are among the critical machining quality indicators [11]. Superior thickness consistency ensures that subsequent thinning and finishing processes operate within stable and controllable parameter ranges, thereby improving yield and reducing costs [12]. Additionally, fiber-reinforced ceramic matrix composites (FRCMCs) have extensive applications in aerospace [13], nuclear engineering [8], and high-end automotive braking systems [14]. Due to the anisotropy and heterogeneity of FRCMCs, the material removal mechanisms and surface integrity characteristics are distinct from those of traditional materials. Consequently, research into high-performance and high-reliability machining technologies for FRCMCs remains a primary focus and a research hotspot.

Abrasive processing technology provides a critical solution to the manufacturing challenges posed by the aforementioned hard, brittle, and difficult-to-machine materials. Abrasive processing covers a wide range of processes and scales from forming to finishing, including diamond wire sawing [15,16], grinding [17], lapping [18], polishing [19], magnetic abrasive finishing [20], abrasive flow machining [21], and superfinishing [22], as well as hybrid or assisted machining methods that incorporate energy fields such as chemical, laser, ultrasonic, and magnetic fields [23]. These techniques demonstrate exceptional machining performance on hard-brittle [24], difficult-to-cut [25], and thermally sensitive materials [26]. However, achieving process controllability and predictability remains challenging due to the coexistence of multi-edge random cutting of abrasives and complex multi-physics coupling effects, including contact mechanics [27], heat generation and dissipation [28], lubrication and cooling [29], and tool wear evolution [30]. Taking diamond wire sawing as an example, the dynamic contact behavior of the flexible tool and the obstruction of coolant transmission within the confined cutting zone can easily lead to instability in the machining state [31,32]. Therefore, achieving precise control over the machining process within a complex and stochastic cutting environment remains a common scientific challenge currently facing the field.

This Special Issue, “Advanced Abrasive Processing Technology and Applications”, aims to address critical challenges in the manufacturing of difficult-to-machine materials by presenting frontier theoretical and practical advancements. A total of ten papers are included, focusing on the latest research findings across four key aspects. First, the sawing techniques for brittle and composite materials are discussed, specifically covering the diamond wire sawing of ceramic micro-channels [33] and the diamond band sawing of resin mineral composites [34]. Second, in the field of precision grinding and honing, the research covers the honing of valve sleeves fabricated from high-hardness martensitic stainless steel [35], creep feed grinding of high-strength steel [36], and the thermal–mechanical coupling analysis of bearing raceway grinding [37]. Third, regarding novel polishing and fluid finishing, the Issue includes findings on phased array ultrasonic cavitation abrasive polishing [38], Halbach array magnetic field-assisted polishing [39], discontinuous film superfinishing [40], and the rheological modeling of abrasive flow machining (AFM) [41]. Finally, the Special Issue emphasizes the importance of process intelligence through a comprehensive review of optimization algorithms [42]. These advanced theories and applications provide valuable insights into high-efficiency, high-quality, and controllable abrasive processing technologies.

## 2. Overview of Published Articles

### 2.1. Sawing Processes for Hard-Brittle and Composite Materials

Diamond wire and band sawing technologies are widely applied in machining hard-brittle and composite materials due to their distinct advantages of low kerf loss and the capability to fabricate precise micro-features. Addressing the challenge of machining hard and brittle ceramic materials for use as catalyst supports in methanol steam reforming (MSR) microreactors, Li et al. [33] employed diamond wire sawing technology to fabricate microchannels with a periodic corrugated microstructure. The effects of wire speed and feed speed on the amplitude and period of the microstructures were investigated through single-factor experiments. The results indicated that, after applying the catalyst via a multiple impregnation method, the periodic corrugated microstructure significantly enhanced the loading strength of the catalyst, as verified by a strong wind purge experiment. Furthermore, application tests in a microreactor demonstrated effective hydrogen production performance. This work provides positive guidance for advancing the use of ceramic catalyst supports in hydrogen energy.

Considering the high damping properties and corrosion resistance but overall brittleness of Resin mineral composite (RMC), Sun et al. [34] investigated the feasibility of machining this material using a diamond band saw. Sawing experiments were conducted to characterize the sawing force and analyze the resulting surface morphology. The results showed that the feed force remained within the range of 3.5~5.5 N with a relatively low tangential force. While the distribution of resin mineral components had little impact on the average force, it significantly increased the fluctuation of the lateral force (by up to 94.86%). Maintaining a constant ratio of sawing speed to feed speed produced consistent surfaces with uniform strip scratches. However, a step structure with a height of approximately 10 μm was observed at the interface. The significance of this study lies in providing a new idea for the precision processing of RMC and promoting the wider application of RMC components in advanced instruments

### 2.2. Precision Grinding and Honing Technology

Precision grinding and honing are pivotal for ensuring the dimensional accuracy and surface integrity of critical industrial components. Addressing the challenges of inefficiency and instability in machining high-hardness martensitic stainless steel valve sleeves, Su et al. [35] conducted a comparative study on the performance of electroplated and sintered oilstones. The influence of different process parameters on processing efficiency and surface quality was systematically explored. The results indicated that electroplated oilstones achieved a material removal rate 2.5 times higher than sintered oilstones; the latter yielded superior surface quality. To reconcile this, the researchers proposed a novel sequential honing strategy—utilizing electroplated oilstones for rapid material removal followed by sintered oilstones for surface finishing—which successfully enhanced overall efficiency by 1.6 times. This study is significant, as it demonstrates the broad application prospects of electroplated oilstones and provides a practical, optimized solution for high-efficiency precision machining of critical hydraulic components.

To mitigate the issues of excessive wheel wear and thermal damage in the creep-feed grinding of high-strength steel, Yin et al. [36] conducted a comparative investigation using SG and CBN abrasive wheels. The study scrutinized grindability parameters—including grinding force, temperature, and specific energy—while systematically analyzing surface integrity factors, such as morphology, roughness, residual stress, and hardness. The findings revealed that thermal damage, manifesting as oxidative discoloration, hardness reduction, and residual tensile stresses, occurs when instantaneous temperatures exceed the phase transition temperature of steel. Notably, the superior hardness and thermal conductivity of CBN abrasives proved more effective than SG grits in suppressing grinding burns. Specifically, the CBN wheel reduced grinding-specific energy by approximately 33% compared to the SG wheel and maintained surface roughness below 0.8 µm. This research concludes that reducing heat generation and enhancing evacuation are essential for burn-free grinding, providing valuable technical guidance for the high-quality precision manufacturing of high-end steel components.

Cheng et al. [37] established a finite element simulation model focused on the inner raceway of tapered roller bearing outer rings. This study aimed to predict the thickness of the grinding-affected layer by simulating the grinding temperature field. The results demonstrated that the maximum grinding temperature concentrates near the center of the grinding arc at the thin end edge of the raceway. Furthermore, the simulations revealed a direct correlation: reducing workpiece speed and grinding depth significantly lowers peak temperatures, thereby decreasing the thickness of the dark-affected layer or preventing its formation entirely. The work provided theoretical guidance and an experimental reference for grinding the raceway of tapered roller bearings.

### 2.3. Novel Polishing and Flow Finishing Methods

Achieving ultra-smooth surfaces on complex geometries requires overcoming the limitations of rigid tools, necessitating the use of flexible media and multi-field assistance. The following articles explore innovative finishing techniques, including ultrasonic cavitation, magnetic field assistance, and rheological modeling, to enhance surface quality and uniformity.

Dai et al. [38] proposed a novel ultrasonic cavitation abrasive flow method based on phase control technology. By integrating ultrasonic phase control theory with Hertzian contact theory, a material removal rate model was established, and COMSOL Multiphysics (version 6.1) simulations were utilized to examine the effects of transducer frequency and spacing on acoustic field distribution and cavitation domain size. The results showed that optimal polishing performance is achieved at a frequency of 21 kHz and a spacing of 100 mm. Experimental results confirmed that polishing efficiency peaks within the first 30 min and stabilizes after 60 min. This research proved the viability of phase control technology in achieving uniform material removal for complex internal surfaces.

Aiming to extend the service life of titanium alloy components by minimizing surface defects, Qin et al. [39] employed a Halbach array-assisted magnetic abrasive polishing method. The study began with simulating and verifying the magnetic field distribution to characterize the specific Halbach array used. Subsequently, polishing experiments were conducted to investigate the evolution of shear force and surface roughness over time, as well as the changes in surface morphology. Furthermore, a response surface model was established to determine optimal process parameters for the maximum shear force and the minimum surface roughness. The results demonstrated that a maximum shear force of 6.11 N was achievable with a tool speed of 724 rpm, a 0.5 mm gap, and 200 μm abrasive particles. A minimum surface roughness of 88 nm was attained at a tool speed of 898 rpm, a 0.52 mm gap, and 160 μm abrasive particles. This work provides optimized parameter sets for balancing material removal force and surface finish quality in magnetic abrasive polishing of titanium alloys.

To enhance surface finishing processes, Tandecka et al. [40] developed innovative abrasive tool prototypes featuring non-continuous abrasive films with discontinuous surface carriers and abrasive layers. Four distinct films with varying nominal grain sizes were fabricated to rigorously evaluate the versatility and efficacy of these prototypes. The results indicated that the incorporation of carrier irregularities plays a significant role in the finishing process, leading to simultaneous improvements in material removal efficiency and surface quality. Specifically, longitudinal discontinuities were found to facilitate the faster removal of material irregularities while reducing the risk of deep surface scratches. Furthermore, the work showed that integrating carrier irregularities with additional oscillatory tool motion holds promise for further enhancing surface quality. This study advances the understanding of abrasive machining mechanisms and offers valuable insights for optimizing abrasive tool designs to achieve superior surface finishing.

Addressing the challenges of non-uniformity and dimensional control in abrasive flow machining (AFM) caused by the complex shear viscosity and wall slip behavior of the media, Peng et al. [41] established a comprehensive framework that combines theoretical foundations with experimental measurement. Utilizing capillary flow, a novel compensation strategy was incorporated into the Mooney method to counter entrance pressure drop effects. This enhanced approach serves as a promising alternative to the conventional Cox–Merz empirical rule, enabling precise characterization of wall slip behavior and shear viscosity, particularly at elevated shear rates. The results indicated that the abrasive media exhibits a Navier nonlinear wall slip. Furthermore, the developed tailored constitutive model and slip model achieved high predictive accuracy with a MAPE not exceeding 6.9%, validating the robustness of the proposed framework for modeling fully developed capillary flow. This study provides a robust foundation that supports authentic modeling and accurate material removal predictions.

### 2.4. Optimization Algorithms and Process Intelligence

As manufacturing moves toward intelligence and sustainability, optimization algorithms play an increasingly important role in parameter optimization during the machining process. Song et al. [42] provided a systematic review of their application and developmental trends within practical engineering production. The study focused on mature methods such as the response surface method, genetic algorithm, Taguchi method, and particle swarm optimization. While these algorithms are extensively used to optimize targets such as surface roughness, cutting force, and subsurface damage, the study identified that current research has reached a plateau of maturity, necessitating the integration of newer approaches like machine learning. The conclusions highlight that real-world engineering often involves complex multi-objective optimization challenges with intricate constraints, where methods like weighting can consolidate multiple objectives into a unified target. Looking forward, the authors predict that future research will pivot toward deep reinforcement learning and digital twinning to enable intelligent manufacturing. The authors emphasize three key trends: enhancing adaptation and self-optimization capabilities for dynamic parameter adjustment; addressing complex systems to meet green manufacturing goals; and promoting algorithm fusion to create hybrid strategies that drive the industry toward greater efficiency and sustainability.

## 3. Conclusions

The articles published in this Special Issue focus on the advancements in abrasive processing technologies and their diverse applications in addressing critical manufacturing challenges. Numerous innovative methodologies and machining process strategies have been proposed, primarily across four specific domains. First, research on diamond wire and band sawing has advanced the application of sawing technologies in the processing of micro-structured ceramics and composite materials [33,34]. Second, in the field of precision grinding and honing, advancements are achieved in the honing of high-hardness martensitic stainless steel valve sleeves [35], the precision grinding of high-strength steel [36], and the thermal–mechanical coupling analysis of bearing raceways [37]. Third, regarding novel polishing and finishing, this Special Issue highlights breakthroughs in phased array ultrasonic cavitation abrasive flow polishing [38], Halbach array magnetic field-assisted abrasive particles polishing [39], discontinuous abrasive films superfinishing [40], and the rheological modeling of abrasive flow machining [41]. Finally, the importance of process intelligence is emphasized through a comprehensive review of optimization algorithms [42]. These studies have promoted the development and expanded the applications of advanced abrasive processing technologies, while simultaneously providing new machining strategies for various difficult-to-machine materials. It also provides theoretical and technical support for developing high-efficiency, high-quality, and intelligent abrasive processing systems.
